# Modeling the formation and dynamics of cortical waves induced by cholinergic modulation

**DOI:** 10.1186/1471-2202-16-S1-P304

**Published:** 2015-12-18

**Authors:** James P Roach, Eshel Ben-Jacob, Leonard M Sander, Michal R Zochowski

**Affiliations:** 1 Neuroscience Graduate Program, University of Michigan, Ann Arbor, MI, 48109, U.S.A; 2 School of Physics and Astronomy, Tel-Aviv University, Tel Aviv, 69978, Israel; 3 Center for Theoretical Biological Physics, and Department of Biochemistry and Cell Biology, Rice University, Houston, TX, 77005, USA; 4 Department of Physics & Center for Studies of Complex Systems, University of Michigan, Ann Arbor, MI, 48109, USA; 5 Biophysics Program, University of Michigan, Ann Arbor, MI, 48109, USA

## 

**Figure 1:**
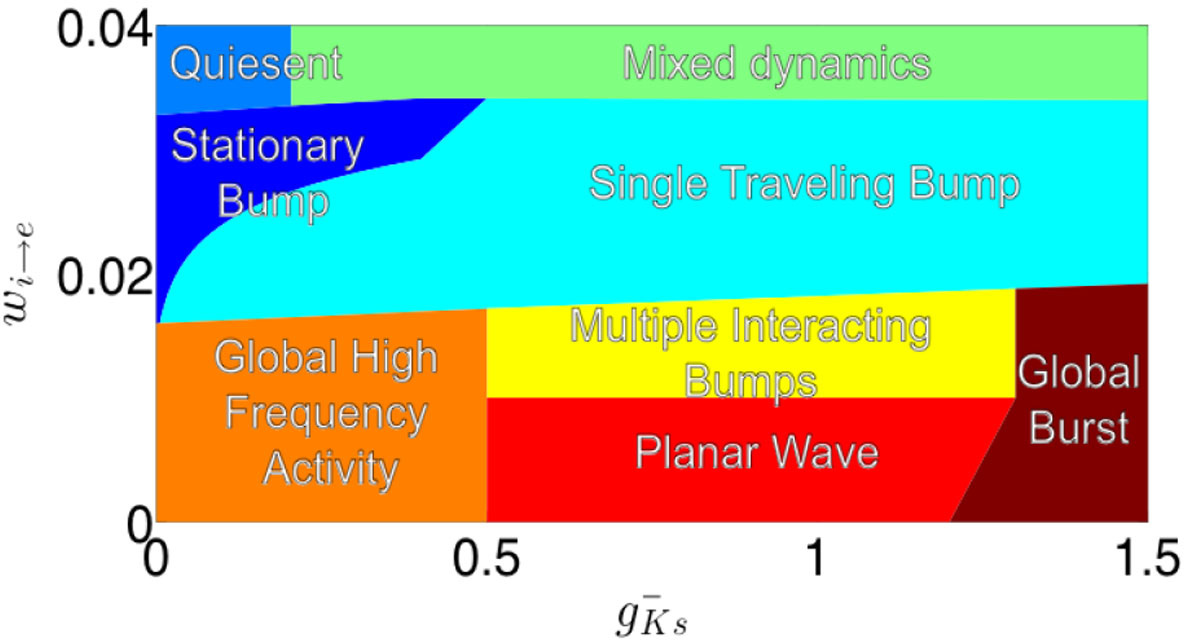
**An illustration of the dynamics sampled by scannig inhibitory strength,(w_i e_), and g_Ks_**. In this model g_Ks _is increased to simulate decreasing ACh levels. In a general sense, the spatial scope of activity is determined by the excitatory/ inhibitory balance, and the temporal scope of activity is determined by the strength of SFA.

States of arousal, or consciousness with the brain are regulated largely by the neurotransmitter acetylcholine (ACh). Specifically, ACh is likely responsible for the transition between slow wave sleep (SWS; where ACh is absent) and rapid eye movement sleep or waking states (where ACh is high). Patterns of neural activity within the cerebral cortex corresponding to these states are markedly different. During SWS there are traveling waves of intense activity in the cortex while in other states locally organized stationary patterns occur [1]. From a functional perspective, stationary patterns are likely to be important for working memory and attention dynamics while traveling waves could lead to synaptic renormalization [2]. The mechanism for how changes on the cellular level are translated to patterns on the network level is not understood. In this work we give a model for the action of ACh on a network of neurons of the Hodgkin-Huxley type with a current that is regulated by ACh that induces spike-frequency adaptation (SFA) [3]. The cells are coupled in a center-surround scheme. When SFA is minimal (such as in waking or REM sleep state, high ACh) patterns of activity are localized and easily pinned to regions defined by enhanced recurrent excitation. Increasing the level SFA is present (by increasing ACh), traveling waves of activity naturally arise. Depending on the strength of inhibitory coupling within the network, SFA is able to induce a wide variety of dynamical regimes (Figure 1). We present a detailed mechanism that shows that the level of inhibition sets the spatial extent of network activity and that SFA defines the temporal scope, which is directly modulated by ACh in the model. These model calculations give unique insights into the role and significance of ACh in determining patterns of cortical activity and functional differences arising from these patterns.
